# Salt and Health: Survey on Knowledge and Salt Intake Related Behaviour in Italy

**DOI:** 10.3390/nu12020279

**Published:** 2020-01-21

**Authors:** Paola Iaccarino Idelson, Lanfranco D’Elia, Giulia Cairella, Paola Sabino, Luca Scalfi, Alessandra Fabbri, Ferruccio Galletti, Francesca Garbagnati, Lillà Lionetti, Gaetana Paolella, Paolo Simonetti, Pasquale Strazzullo, on behalf of the SINU-GIRCSI Working Group

**Affiliations:** 1 Department of Clinical Medicine and Surgery, “Federico II” University of Naples Medical School, Via Sergio Pansini n.5, 80131 Napoli, Italy; lanfranco.delia@unina.it (L.D.); dottoressasabino@gmail.com (P.S.); ferruccio.galletti@unina.it (F.G.); francesca.garbagnati@gmail.com (F.G.); pasquale.strazzullo@unina.it (P.S.); 2 Prevention Department, ASL Rome 2, 00100 Rome, Italy; giuliacairella@gmail.com; 3 Department of Public Health, “Federico II” University of Naples Medical School, 80100 Naples, Italy; scalfi@unina.it (L.S.); gaetana.paolella@gmail.com (G.P.); 4 Department of Public Health, AUSL IRCCS Reggio Emilia, 42121 Reggio Emilia, Italy; alessandra.fabbri@ausl.re.it; 5 Department of Chemistry and Biology, University of Salerno, 84084 Fisciano, Italy; lionetti@unisa.it; 6 Department of Food, Environment and Nutritional Science, University of Milan, 20019 Milan, Italy; 7 Working Group for Reduction of Salt Intake, Italian Society of Human Nutrition (SINU), 20126 Milan, Italy

**Keywords:** salt consumption, knowledge, behaviour

## Abstract

Background and aim: Excess sodium intake is a recognised causal factor of hypertension and its cardiovascular complications; there is however a lack of practical instruments to assess and monitor the level of knowledge and behaviour about dietary salt intake and to relate these factors to the population general dietary habits. Methods and Results: A self-administered questionnaire was developed to assess the salt and health related knowledge and behaviour of the Italian population through an online survey. A sample of 11,618 Italian participants completed the questionnaire. The degree of knowledge and the reported behaviour about salt intake were both found to be related to age, gender, home region, level of education and occupation. There was a significant interrelation between salt knowledge and behaviour and both were significantly and directly related to the degree of adherence to a Mediterranean-like dietary pattern. A hierarchical evaluation was also made of the relevance of any single question to the overall assessment of knowledge and behaviour about salt intake. Conclusions: The study population overall appeared to have a decent level of knowledge about salt, but a less satisfactory behaviour. Our findings point to social inequalities and young age as the main factors having a negative impact on knowledge and behaviour about salt intake as part of generally inadequate dietary habits. The degrees of knowledge and behaviour were significantly and directly interrelated, confirming that improving knowledge is a key step for behavioural changes, and suggesting that educational campaigns are crucial for the implementation of good practices in nutrition.

## 1. Introduction

Noncommunicable diseases (NCDs) are the main cause of death worldwide [1] and cost-effective strategies are needed to reduce their burden. 

Elevated sodium intake is a causal factor of NCDs, with particular regard to hypertension and its cardiovascular complications [2]. Data on sodium intake in most populations show that salt consumption is much higher than physiologically needed [3] and recommended by WHO [4]. Reducing salt intake was shown to reduce blood pressure [5], to lower the incidence of hypertension [6] and accordingly to decrease the burden of cardiovascular events [7,8,9].

For these reasons, public health specialists are engaged in the development of effective campaigns for behavioural modifications, particularly needed for salt intake reduction [10]. Noteworthy, plant-based nutritional models such as the Mediterranean Diet (MD), which are known to reduce the risk of cardiovascular disorders [11], are also moderate in their salt content, because of their richness in vegetable, fruit and non-processed foods, which have a lower sodium content compared with the process foods more abundant in the Western model [12].

According to the “food literacy” model [13], knowledge is essential to put in practice healthy diets [14]. Food literacy is a collection of the inter-related knowledge, skills and behaviours required to plan, select, prepare and eat foods to meet physiological needs. 

With respect to salt consumption, reliable estimates about the habitual salt intake in Italy have been made available by the MINISAL-GIRCSI study (a study conducted by the Interdisciplinary Working Group for Reduction of Salt Intake of the Ministry of Health) both for the adult population [15,16] and for children and adolescents [17], showing high average values in all age groups, even in hypertensive patients followed at specialised hypertension centres [18]. In particular, the MINISAL-GIRCSI study highlighted that sodium intake is more than recommended (i.e., >5 g or 85 mmol/day) in 97% of the men and 87% of the women in a country where the prevalence of hypertension is 53.7% in men and 40.3% in women (http://www.cuore.iss.it/fattori/CuoreData.asp). However, there is a lack of systematic and reliable information about the degree of knowledge and behaviour of the Italian population about salt intake and related health issues. Therefore, the aim of the present study was to evaluate knowledge and behaviour of the Italian general population with respect to salt and to explore their interrelation and their relationship to MD adherence, using a user-friendly freely accessible online survey.

## 2. Methods

### 2.1. Development of a Salt Literacy Questionnaire

We developed a self-administered questionnaire to assess the salt-health related knowledge and the behaviour concerning salt consumption of the survey participants. The questionnaire includes all multiple-choice close-ended questions to guarantee standardisation and rapid electronic evaluation; it could be completed in an electronic format online; every question had to be answered to be able to pass to the next one, allowing immediate automatic transfer to an EXCEL file and subsequent statistical analyses.

The questionnaire was developed by PSt and PSa, with the collaboration of LD, CG and GP as follows:

The initial step was the search of the scientific literature on knowledge about salt and health, salt behaviour, salt consumption, salt dietary sources, and tools to assess MD adherence. The questions on knowledge and behaviour were based on the authors’ knowledge and professional experience about the discretionary and non-discretionary dietary sources of salt intake, food composition and population dietary habits [19,20,21,22,23]. To keep the questionnaire as short as possible, only four questions on MD adherence were chosen as the crucial ones to assess this dietary pattern in most published questionnaires [24,25,26]. A question on extra-virgin olive oil was added, because its consumption reflects a particular attention to the quality of food in Italy. 

The second step was a literature search on questionnaire development and food literacy [13,14,27,28]. This brought to the first version of the questionnaire, phrased in a comprehensible language, avoiding the use of technical-scientific terms, and tested on a pilot sample of 300 adolescents and adults with different educational and occupational levels (exploratory factor analysis). The participants to the pilot study recorded the time taken to fill the questionnaire, the intelligibility of the questions and their relative answers and any difficulties in answering the questions.

A final discussion among the experts and the adaptation to the results of the pilot study brought to the final version of the questionnaire (see Appendix A).

The questionnaire is anonymous and includes a first introductory part exploring demographic and social features of the survey participant (gender, age, home region, level of education and type of occupation). The core of the questionnaire includes three sections: (1) knowledge (5 questions, 14 items); (2) behaviour (10 questions, 17 items); and (3) MD adherence (4 questions, 4 items). Three additional questions concerned iodised salt consumption, the quality of the questionnaire itself, and the usefulness of the questionnaire in raising the respondent’s consciousness on the importance of reducing salt consumption (4 items). Upon completion of the questionnaire, the respondent obtained a synthetic judgement on his/her knowledge and behaviour about salt. 

A 2-, 3- or 4-point Likert scale was used for each question and scores were imputed to the different possible answers in the range 0–2. The knowledge section score could span from 0 to 28, the behaviour score from 0 to 26 and the MD adherence score from 0 to 8. The study was approved by the Scientific Council of the SINU, CD 02/2016.

### 2.2. Data Collection

The questionnaire has been available on the Italian Society of Human Nutrition (SINU) website since June 2016 and its completion was encouraged in different settings (educational events, the annual Salt Awareness Week campaign, secondary schools and working place canteens). Moreover, it was spontaneously completed by visitors of the SINU website (www.sinu.it). No registration was needed to complete the questionnaire.

For the purpose of the present study, the data collection was stopped on 1 March 2018.

### 2.3. Statistical Analysis

#### 2.3.1. Exploratory Factor Analysis and Internal Consistency

For the analysis of the first version of the questionnaire in the pilot sample, Bartlett’s test of sphericity and Kaiser–Meyer–Olkin (KMO) were used to assess whether data were suitable for exploratory factor analysis. To detect the number of potential underlying factors, the following criteria were applied: eigenvalues > 1, scree plot, factor loadings > |0.40| and plausibility of the factors in terms of their substantive meaning. Varimax was used for factor rotation. In addition, the Cronbach’s α-coefficient was used to assess internal consistency of test scores reliability.

#### 2.3.2. Evaluation of Knowledge and Behaviour about Salt Intake

The score distribution for both the knowledge and the behaviour sections was not normal (Kolmogorov–Smirnov test: *p* < 0.05). Thus, non-parametric Mann–Whitney or Kruskal–Wallis tests were used to assess the relationship between a single question (or a questionnaire section) score and the various sociodemographic indicators. Spearman’s rank correlation was used to assess both the interrelation between different section scores and the relevance of each single question with respect to the total variance of the knowledge and the behaviour scores. A multivariate linear regression analysis was used to determine the independent effect of each sociodemographic indicator on knowledge and behaviour scores.

Results are expressed as percentage or mean and standard error (SE) or median and 25th–75th percentile as appropriate. Two-sided *p* values < 0.05 were considered statistically significant. The statistical analysis was carried out using the SPSS for Windows, version 23 (SPSS Inc., Chicago, IL, USA).

## 3. Results

### 3.1. Exploratory Factor Analysis and Internal Consistency

The preliminary examination of the questionnaire showed the suitability of the sample for factor analysis (knowledge: KMO = 0.77, Bartlett’s test of sphericity: *p* < 0.001; behaviour: KMO = 0.83, Bartlett’s test of sphericity: *p* < 0.001). In consideration of the pre-defined criteria to detect potential underlying factors, three and five factors emerged which accounted for 60% of the variance observed in the knowledge and the behaviour sections, respectively. Therefore, we divided the knowledge section into three subsections ((a) general knowledge: Questions 1–2 and 4; (b) knowledge about food salt content: Question 3; and (c) ability of reading nutritional labels: Question 5) and the behaviour section into five subsections ((a) reading nutritional labels: Question 5; (b) actions to lower salt intake: Question 6; (c) consumption of high salt content: Questions 7–8; (d) discretionary salt use: Questions 9–12; and (e) individual taste: Questions 13–14). Question 5 was counted in both knowledge and behaviour sections because, while it does concern a practical behaviour, such behaviour is influenced by the participant degree of knowledge about the average sodium content of each given food category.

Finally, the analysis of internal consistency indicated that the questionnaire had a good ability to measure both the level of knowledge (Cronbach’s α-coefficient: 0.71, 14 items) and behaviour about salt consumption (Cronbach’s α-coefficient: 0.81, 17 items). These reliability measures did not vary substantially by the exclusion of any single-item value.

### 3.2. Sample Characteristics

In total, 11,618 questionnaires were available and used for statistical analysis. The sociodemographic characteristics of the participants are summarised in Table 1. Overall, 6683 women (57.5% of the entire sample) and 4935 men (42.5%) completed the questionnaire: more than half of them lived in northern Italy (55.5%), age distribution was similar among the different categories and 3/4 of respondents had completed high school or university. Over 40% were unemployed, being homemakers or students, whereas 26.9% were blue- or low-level white-collar workers, or farmers. 

### 3.3. Knowledge Regarding Salt Intake

**Figure 1:**
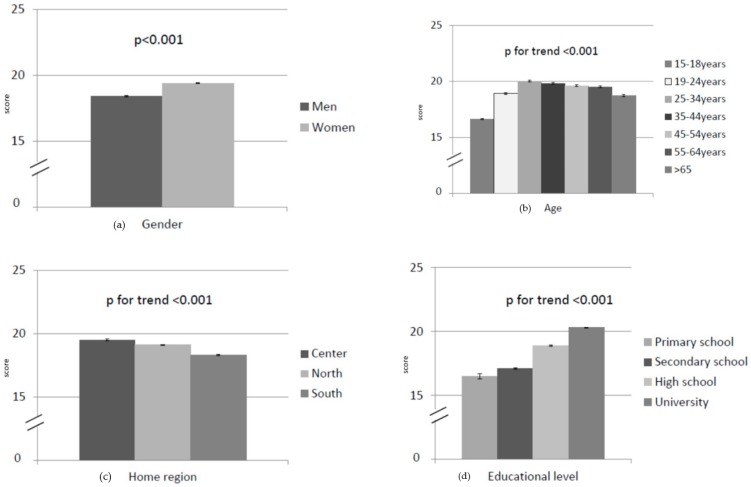

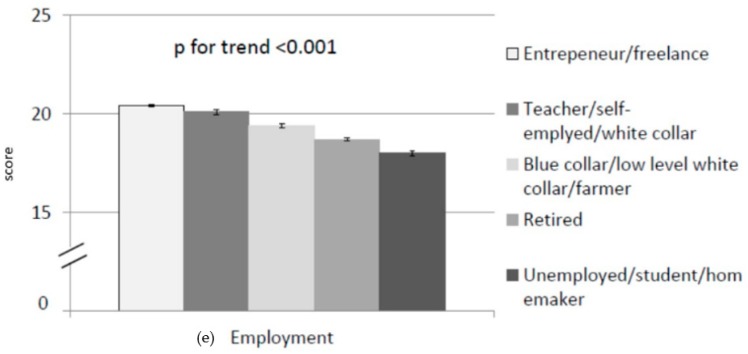
Knowledge about salt intake in relation to sociodemographic factors: (**a**) gender; (**b**) age; (**c**) home region; (**d**) educational level; and (**e**) employment. Results are expressed as mean ± standard error (*n* = 11,618).

The results on knowledge about salt as related to sociodemographic factors are reported in Figure 1 and Table 1. The minimum individual score was 4 and the maximum 28. Women had a significantly higher knowledge score than men, whereas adolescents (15–18 years old) had the lowest knowledge score, similar to people from southern Italy and to less educated people. Entrepreneurs and freelancers had significantly higher knowledge levels compared with unemployed, students and homemakers (*p* < 0.01). The multivariate linear regression analysis indicated that gender, age, level of education and occupation independently affected the total knowledge score (*p* < 0.05), while this was not the case for the home region indicator (*p* > 0.05).

### 3.4. Behaviour about Salt Intake

**Figure 2:**
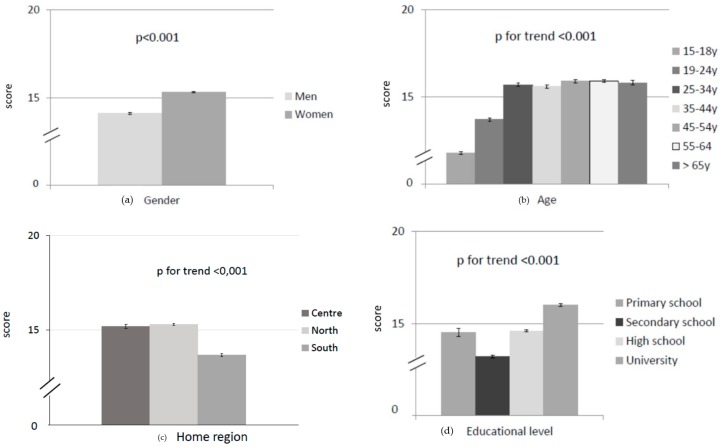

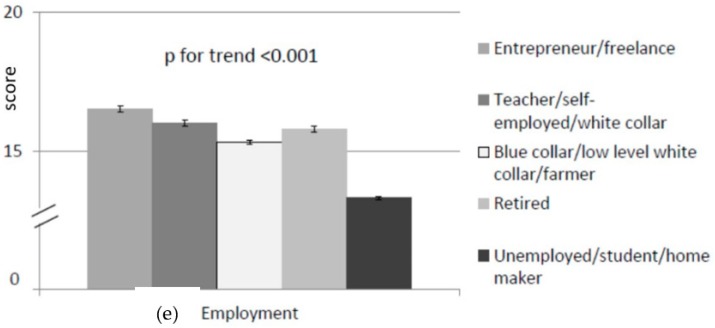
Behaviour about salt intake in relation to sociodemographic factors: (**a**) gender; (**b**) age; (**c**) home region; (**d**) educational level; and (**e**) employment. Results are expressed as mean ± standard error (*n* = 11,618).

Data on behaviour about salt intake are summarised in Table 1 and Figure 2. The individual scores ranged between 0 and 26. Again, women had a significantly higher score than men (*p* < 0.01), and adolescents had a significantly lower score than adults (*p* < 0.01). Again, people from southern Italy had the lowest behaviour score (*p* < 0.01) and people with a university degree had a better behaviour than people with only a primary school diploma (*p* < 0.01). Finally, all types of workers and employees, even if retired, had a significantly better behaviour than unemployed, students and homemakers (*p* < 0.01). The multivariate linear regression analysis indicated that all sociodemographic indicators independently affected the total behaviour score (*p* < 0.05).

### 3.5. Mediterranean Diet Adherence

**Figure 3:**
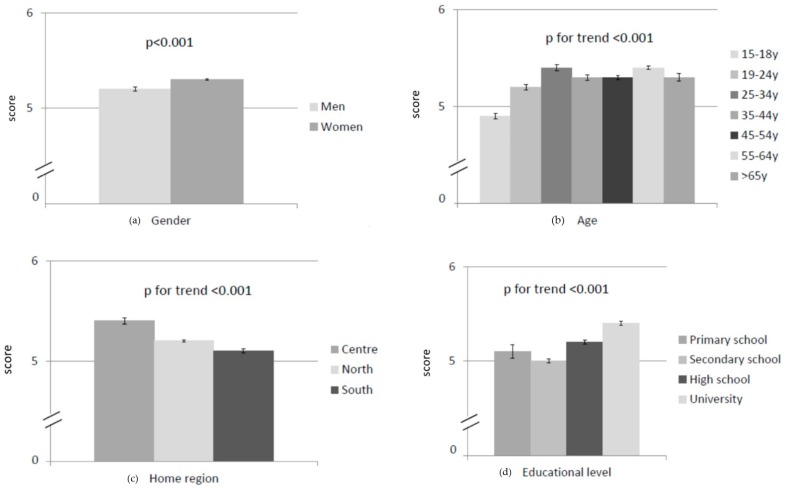

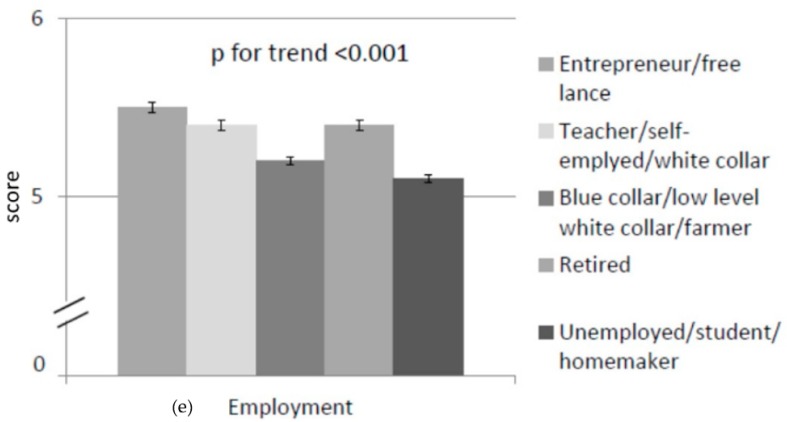
Adherence to the Mediterranean Diet in relation to sociodemographic factors: (**a**) gender; (**b**) age; (**c**) home region; (**d**) educational level; and (**e**) employment. Results are expressed as mean ± standard error (*n* = 11,618).

Results on the adherence to MD are shown in Table 1 and Figure 3. The minimum individual score was 1 and the maximum 8. MD adherence was slightly but significantly better in women than in men (*p* < 0.01), in adults than in adolescents (*p* < 0.01), in people from northern and central Italy compared with people from the south (*p* < 0.01), and in people with a university degree compared with those with only a primary school diploma. Finally, all types of workers, even if retired, had a significantly higher level of MD adherence than unemployed, students and homemakers (*p* < 0.01). The multivariate linear regression analysis indicated that gender, age, level of education and occupation independently affected the total MD adherence score (*p* < 0.05), while this was not the case for the home region indicator (*p* > 0.05). 

### 3.6. Relationship among Knowledge, Behaviour and MD Adherence

**Figure 4:**
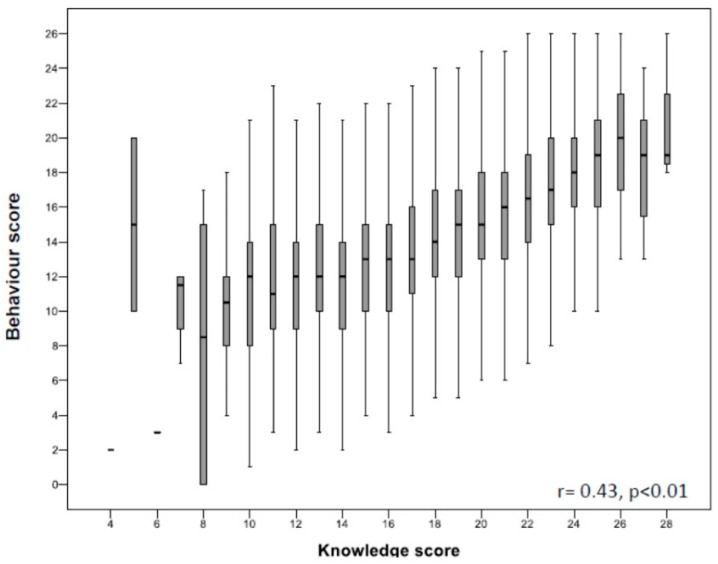
Relationship between knowledge and behaviour about salt consumption. Results are expressed as median (25th–75th percentile).

As shown in Figure 4, there was a significant and positive correlation between salt knowledge and behaviour scores (*r* = 0.42; *p* < 0.001), suggesting that being more informed induced better everyday practices. Moreover, both knowledge and behaviours were significantly related to MD adherence (*r* = 0.23 and *r* = 0.29, respectively, *p* < 0.001).

### 3.7. Relevance of Single Questions

In general, the greater the variability in the reply to any given question the greater was its overall relevance. As to the knowledge section (see Appendix A), the most relevant questions were: Questions 1 (*r* = 0.41), 3a (*r* = 0.43), 3b (*r* = 0.50), 3d (*r* = 0.48), 3g (*r* = 0.48) and 3h (*r* = 0.51).

As to the behaviour section, the most relevant questions were: Questions 5 (*r* = 0.63), 6 (*r* = 0.87), 9 (*r* = 0.50), 11 (*r* = 0.55) and 12 (*r* = 0.50). 

### 3.8. Additional Information

Additional information came from the last three questions (Appendix A): the reply to the question that explored the perception people had of their habitual salt consumption (Question 19) was positively related to the behaviour score with a significant linear trend (*r* = 0.38, *p* < 0.01). Half of our study population regularly used iodised salt (Question 20) and almost everybody (97.0%) was aware of the relationship between excess salt consumption and health problems (Question 21a). Finally, more than 80% of the participants declared that completing the questionnaire was useful to raise their own awareness on the importance of reducing salt consumption (Question 21d).

## 4. Discussion

The impact of habitual salt intake on blood pressure and cardiovascular outcomes has been known since the late 1950s, yet systematic actions by governments and public health institutions to improve the population awareness and to generate a lower sodium intake have lagged behind. Although some questionnaires aiming at the estimation of sodium intake were elaborated already in 1943 [29] and the need to retrieve information about the general population’s behaviour was perceived already in the 1950s [30], a questionnaire on knowledge and behaviour about salt consumption has been lacking so far in Italy, while similar surveys have recently been conducted in other European countries [31].

The completion of the questionnaire by almost 12,000 Italians has thus filled an important gap in our understanding of the degree of knowledge and behaviour of the Italian population on this major nutritional issue. The analysis of the replies provided several pieces of novel information. The respondents overall appear to: (a) have a decent level of knowledge about salt; (b) be aware of the negative effects of excess salt intake on health; (c) know the maximum level of salt intake recommended by public institutions; and (d) are reasonably well informed about the salt content of most foods they consume. Nevertheless, remarkable differences in the degree of knowledge were detected in relation to sociodemographic markers, indicating a great potential for improvement, in particular among adolescents, less educated people and people with low level of employment/unemployed. Among these subgroups, access to adequate information may be insufficient and/or these conditions may be associated with a relative lack of interest in acquiring information about this problem and perhaps about nutritional issues in general [32]. Moreover, among these categories, the consumption of ready-to eat food and ultra-processed foods (often rich in salt) is largely diffused, for cultural, economic and political reasons [33]. 

The behaviours of the survey participants were in general less satisfactory in comparison with their degree of knowledge. The replies to a few key questions suggested inconsistent behaviours; for example, more than 2/3 of the participants stated they avoided or had reduced the consumption of salt-rich foods and they did not add salt while cooking or eating; nevertheless, much less than 50% read the salt content on nutritional labels and bought lower salt food products. Moreover, while eating out, only 10% of the respondents asked for lower salt alternatives. Only a small minority declared to add no salt to meat, fish or fried foods, both while cooking and at the table.

These negative behaviours were particularly common among adolescents, in line with the results of other surveys in adolescents’ populations, which show that many nutritional guidelines are not met by this specific age group, both in Italy [34] and in Europe [35].

It is noteworthy that the degrees of knowledge and behaviour were significantly and directly interrelated, confirming that improving knowledge is a key step for behavioural changes [14], and suggesting that educational campaigns are crucial for the implementation of good practices in nutrition.

The questionnaire did not aim to assess and did not allow to analyse in depth participants’ MD adherence. Nevertheless, it provided sufficient information to conclude that the survey participants had on average a fair level of MD adherence, again with worse performance among adolescents, in accordance with other recent surveys [36,37,38]. An interesting novel finding was that both the degree of knowledge and behaviour about salt intake were significantly correlated with the level of MD adherence, indicating that nutritional information and good eating habits tend to go hand in hand. This finding also confirms that MD adherence is quite compatible with a relatively low salt intake [12] and that promoting MD adherence may also be a good tool to reduce salt consumption.

The analysis of the relevance of each single question with respect to the total variance in the participants’ knowledge and behaviour allowed to understand what are the crucial issues to pay attention in epidemiological surveys. Some notions were shared by the large majority of the survey population (e.g., the low salt content of fruits, vegetables or milk); however, for others, knowledge and behaviours were much less straightforward. These are obviously key elements on which to concentrate future educational efforts.

The SINU salt questionnaire also aimed at raising the participants’ awareness on the importance of reducing salt consumption. We believe that this goal was achieved in two ways: (1) by focusing the subject’s attention on these specific questions; and (2) by providing the respondents with a final score on knowledge degree and type of behaviour about salt intake. The latter had the intent to reinforce motivation in those who received higher scores and stimulate those with worse performance to enhance their knowledge and improve their nutritional behaviour.

Our study had several strengths and limitations. The strengths are the large number of survey participants, their wide geographical distribution with all the Italian regions involved, the very large age distribution and the relatively short time of data collection. Moreover, the results of specific statistical tests supported the validity of the questionnaire and of the survey, suggesting that the information obtained was sufficiently reliable. We also acknowledge a few study limitations: (i) the SINU website is probably visited to a larger degree by people having interest in the field of nutrition (although this selection bias with respect to external validity may be partially offset by the large number of participants); and (ii) in some educational events involving a few hundred people, the questionnaire was compiled under the supervision of an expert nutritionist (who however did not influence the replies provided to the single questions). 

## 5. Conclusions

Considering the strong need for effective public health interventions aiming at reducing salt consumption, the newly established online questionnaire provided reliable and novel information about knowledge and behaviour concerning salt intake in Italy. The results of the survey suggest that Italians have on average a decent level of knowledge and pay attention to excess salt intake. On the other hand, our results also point to lower educational level and social inequalities as factors that negatively affect both knowledge and behaviour about this major nutritional issue and likewise about nutritional habits in general. The results also point to adolescence as an age category with limited knowledge and poorer behaviour about salt consumption. Further studies are needed to understand how to improve the practical behaviours about salt intake. A synthesis of the state of the art on the topic and of the conclusions of this work can be found in Table 2.

We believe that the SINU online salt questionnaire may be a useful instrument to monitor changes in knowledge and behaviours about salt intake. It could also be used in other countries (both Mediterranean and non-Mediterranean ones), making a few adaptations in the first section by including the foods most typical for each country. 

## Figures and Tables

**Table 1:** Sociodemographic characteristics and mean (standard error) + medians (25th–75th percentile) of knowledge, behaviour and adherence to the Mediterranean Diet (MD) of the study population (*N* = 11,618).

	*N*	%	Knowledge		Behaviour		MD Adherence	
			Mean (SE)	Median (25th–75th Percentile)	Mean (SE)	Median (25th–75th Percentile)	Mean (SE)	Median (25th–75th Percentile)
Total	11,618	100	20.6 (0.03)	19 (17–21)	14.8 (0.04)	15 (12–18)	5.2 (0.01)	5 (4–6)
Gender								
Men	4935	42.5	20.0 (0.05)	20 (18–22)	14.1 (0.06)	14 (11–17)	5.2 (0.02)	5 (4–6)
Women	6683	57.5	21.0 (0.04)	21 (19–23) ^a^	15.3 (0.05)	15 (13–18) ^a^	5.3 (0.01)	5 (5–6) ^a^
Age (years)								
15–18	2164	18.6	18.3 (0.07)	18 (16–20) *	11.8 (0.08)	12 (9–14) *	4.9 (0.03)	5 (4–6) *
19–24	1354	11.7	20.4 (0.08)	21 (18–22)	13.7 (0.11)	13 (11–16)	5.2 (0.03)	5 (4–6)
25–34	1617	13.9	21.6 (0.08)	22 (20–24)	15.7 (0.10)	15 (13–18)	5.4 (0.03)	5 (5–6)
35–44	1483	12.8	21.4 (0.08)	22 (20–24)	15.6 (0.10)	15 (13–18)	5.3 (0.03)	5 (5–6)
45–54	2631	17.1	21.2 (0.07)	21 (19–23)	15.9 (0.09)	16 (13–18)	5.3 (0.02)	5 (5–6)
55–64	2003	17.2	21.1 (0.07)	21 (19–23)	15.9 (0.08)	16 (14–18)	5.4 (0.02)	5 (5–6)
>65	1006	8.7	20.3 (0.10)	21 (19–23)	15.8 (0.13)	16 (13–18)	5.3 (0.04)	5 (5–6)
				*p* for trend < 0.001		*p* for trend < 0.001		*p* for trend < 0.001
Home region								
Centre	1890	16.3	21.1 (0.07)	21 (19–23)	15.2 (0.10)	15 (12–18)	5.4 (0.03)	5 (5–6)
North	6417	55.2	20.7 (0.04)	21 (19–23)	15.3 (0.05)	15 (13–18)	5.2 (0.01)	5 (4–6)
South	3311	28.5	20.0 (0.06)	20 (18–22) *	13.7 (0.08)	13 (11–17) *	5.1 (0.02)	5 (4–6) *
				*p* for trend < 0.001		*p* for trend < 0.001		*p* for trend < 0.001
Level of education completed								
Primary school	289	2.5	17.7 (0.20)	17 (15–20) *	14.5 (0.22)	15 (12–17) *	5.1 (0.07)	5 (4–6) *
Secondary school	2505	21.6	18.8 (0.06)	19 (17–21)	13.2 (0.08)	13 (10–16)	5.0 (0.02)	5 (4–6)
High school	4649	40.0	20.5 (0.04)	21 (19–23)	14.6 (0.06)	15 (12–17)	5.2 (0.02)	5 (4–6)
University	4175	35.9	21.9 (0.04)	22 (20–24)	16.0 (0.06)	16 (13–19)	5.4 (0.02)	5 (5–6)
				*p* for trend < 0.001		*p* for trend < 0.001		*p* for trend < 0.001
Occupation								
Entrepreneur/Freelance	1240	10.7	22 (0.09)	22 (20–24)	16.5 (0.11)	17 (14–19)	5.5 (0.03)	5 (5–6)
Teacher/self-employed/white collar worker	1403	12.1	21.7 (0.08)	22 (20–24)	16.0 (0.10)	16 (14–18)	5.4 (0.03)	5 (5–6)
Blue collar/low level white collar worker/Farmer	3121	26.9	21.0 (0.08)	21 (19–23)	15.5 (0.07)	15 (13–18)	5.2 (0.02)	5 (5–6)
Retired	1135	9.8	20.3 (0.09)	21 (18–22)	15.8 (0.11)	16 (13–18)	5.4 (0.03)	5 (5–6)
Unemployed/Student/Homemaker	4719	40.6	19.6 (0.05)	20 (17–22) *	13.3 (0.06)	13 (10–16) *	5.1 (0.02)	5 (4–6) *
				*p* for trend < 0.001		*p* for trend < 0.001		*p* for trend < 0.001

SE, standard error; * reference group; ^a^ vs. Men: *p* < 0.001.

**Table 2:** Synthesis of the state of the art on the topic and of the conclusions of the work.

What is already known on this topic ➢ Salt consumption is much higher than physiologically needed and recommended by WHO. ➢ Reducing salt intake is useful to reduce blood pressure and to lower the incidence of hypertension. ➢ According to the “food literacy” model, knowledge is essential to put in practice healthy behaviours.
What this study adds ➢ The significant and positive correlation detected between salt knowledge and behaviour score implies that being more informed induces better everyday practices. ➢ Younger people and those with low educational level are the categories with more limited knowledge and poorer behaviour about salt consumption. ➢ The survey improved our understanding on the crucial issues to focus on in epidemiological surveys of dietary salt habits.

## Appendix A

**Table A1:** Questionnaire items on knowledge and behaviour about salt consumption and adherence to the Mediterranean Diet. Replies to single questions by response categories [n (%)] and relevance of each question (r) to the overall assessment of the knowledge score and the behaviour score. The questionnaire is available in Italian on www.sinu.it.

	Score	*N*	(%)	r *
**KNOWLEDGE**				
**1. In your opinion, how important is it to keep a low salt intake?**				0.41
Very important	2	8155	70.2	
Quite important	1	3188	27.4	
Not so important	0	275	2.4	
**2. According to nutritional guidelines what is the maximum salt intake for an adult to maintain a healthy diet?**				0.29
2 g/day	1	2837	24.4	
5 g/day	2	8014	69.0	
10 g/day	0	767	6.6	
**3. To the best of your knowledge, how much salt do the following foods contain (please answer all questions):**				
**3a. Standard white bread**				0.43
Little	0	5087	43.8	
Quite a lot	1	5367	46.2	
A lot	2	1164	10.0	
**3b. Pizza**				0.50
Little	0	1790	15.4	
Quite a lot	1	6179	53.2	
A lot	2	3649	31.4	
**3c. Pasta, rice, other grains**				0.23
Little	2	9017	77.6	
Quite a lot	1	2100	18.1	
A lot	0	501	4.3	
**3d. Aged cheeses and cured meats**				0.48
Little	0	692	6.0	
Quite a lot	1	2890	24.9	
A lot	2	8036	69.2	
**3e. Milk**				0.13
Little	0	217	1.9	
Quite a lot	2	1372	11.8	
A lot	1	10,029	86.3	
**3f. Fruit, vegetables, legumes**				0.30
Little	2	10,529	90.6	
Quite a lot	1	799	6.9	
A lot	0	290	2.5	
**3g. Canned foods**				0.48
Little	0	714	6.1	
Quite a lot	1	4776	41.1	
A lot	2	6128	52.7	
**3h. Ready-made sauces**				0.51
Little	0	673	5.8	
Quite a lot	1	3977	34.2	
A lot	2	6968	60.0	
**3i. Meat and fish**				0.31
Little	2	8753	75.3	
Quite a lot	1	2317	19.9	
A lot	0	548	4.7	
**3j. Walnuts, almonds, hazelnuts**				0.30
Little	2	8820	75.9	
Quite a lot	1	2111	18.2	
A lot	0	687	5.9	
**4. In your opinion, how many grams of salt does a single gram of sodium correspond to?**				0.38
1 g/5 g/10 g	0	4920	42.3	
2.5 g	2	6698	57.7	
**BEHAVIOUR**				
**5. Does the information about salt quantity reported on nutritional labels affect your food choices when grocery shopping? ‡**				0.36/0.63
Always	2	1732	14.9	
Sometimes	1	5519	47.5	
Never	0	4367	37.6	
**6. What do you normally do to eat little salt? (please choose as many responses as apply)**				0.87
I avoid eating, or I reduce the consumption of products rich in salt				
NO	0	3471	29.9	
YES	1	8147	70.1	
I buy alternative products, with a low salt content				
NO	0	6560	56.5	
YES	1	5058	43.5	
I read the salt content on the nutritional labels				
NO	0	7238	62.3	
YES	1	4380	37.7	
I had no, or very little salt				
NO	0	2624	22.6	
YES	1	8994	77.4	
I don’t add salt while cooking (or only very little)				
NO	0	4521	38.9	
YES	1	7097	61.1	
I use spices instead of salt in cuisine				
NO	0	4767	41.0	
YES	1	6851	59.0	
I avoid going out to eat out too often				
NO	0	5018	43.2	
YES	1	6600	56.8	
If I eat out I ask for low salt options				
NO	0	10,423	89.7	
YES	1	1195	10.3	
**7. How much bread do you usually eat?**				0.40
I usually eat bread without or with very little salt	2	3600	31.0	
Not more than 3 slices	1	6713	57.8	
More than 3 slices	0	1305	11.2	
**8. How many times per week do you usually eat cheese and/or cured meat?**				0.41
0–2 times/week	2	6587	56.7	
3–4 times/week	1	4163	35.8	
5 or more times/week	0	868	7.5	
**9. When you cook/eat vegetables/legumes, you add salt:**				0.50
Only during preparation OR Only at the table	1	4163	35.8	
Both while cooking and at the table	0	868	7.5	
Neither during preparation nor at the table	2	6587	56.7	
**10. When you eat pasta, rice, other cereals, you add salt:**				0.39
Only during preparation/Only at the table	1	8833	76.0	
Both while cooking and at the table	0	859	7.4	
Neither during preparation nor at the table	2	1926	16.6	
**11. When eating meat or fish, you add salt:**				0.55
Only during preparation/Only on the table	1	9927	85.4	
Both while cooking and on the table	0	500	4.3	
Neither during preparation nor on the table	2	1191	10.3	
**12. When you cook or eat fried food (meat, fish, vegetables, potatoes, etc.), you add salt:**				0.50
Only during preparation/Only at the table	1	8511	73.3	
Both while cooking and at the table	0	990	8.5	
Neither during preparation nor at the table	2	2117	18.2	
**13. Are you thirsty after meals?**				0.44
Never	2	1753	15.1	
Often	1	9073	78.1	
Always	0	792	6.8	
**14. When eating away from home to me food tastes:**				0.49
Flavorless	0	667	5.7	
Normal	1	3991	39.4	
Salty	2	6960	59.9	
**MEDITERRANEAN DIET**				
**15. What do you normally use for dressing? What condiment would you normally use?**				0.32
Extra-virgin olive oil	2	4063	35.0	
Olive oil/Seeds oil	1	7228	62.2	
Butter or lard/Margarine	0	327	2.8	
**16. How many servings of fruit (including nuts), vegetables, legumes do you usually consume per day?**				0.66
a. 3 or less	0	120	1.0	
b. 4–5	1	969	8.3	
c. More than 5	2	10,529	90.6	
**17. How many servings of pasta/rice do you normally have per day?**				0.22
1 or more	2	7578	65.2	
Less than 1	1	4040	34.8	
**18. How many times per day do you usually eat biscuits, sugar sweetened snacks, or other types of sweets?**				0.66
No more than 1	1	5495	47.3	
Less than 1	2	3795	32.7	
More than 1	0	2331	20.1	
**ADDITIONAL INFORMATIONS**				
**19. In conclusion, in your opinion, how much is your daily salt intake, taking into account the foods you buy and the ones you personally cook (including the salt added during preparation and/or after cooking)?**				
Less than 5 g per day		3574	30.8	
Between 5 and 10 g		6738	58.0	
Between 10 and 20 g		1191	10.3	
More than 20 g		115	1.0	
**20. Do you usually use iodised salt for seasoning or cooking?**				
Always		5821	50.1	
Sometimes		3480	30.0	
Never		2317	19.9	
**21. In conclusion, we would like to know your opinion on the questionnaire. In your opinion:**				
Excessive salt intake is a relevant topic for health				
NO		351	3.0	
YES		11,267	97.0	
Are the questions clear and easily understandable?				
NO		916	7.9	
YES		10,702	92.1	
Is this questionnaire useful to understand people’s behaviour on salt consumption?				
NO		1773	15.3	
YES		9845	84.7	
Did this questionnaire make you reflect on your own behaviour regarding salt consumption?				
NO		2054	17.7	
YES		9564	82.3	

* Association between the answers provided to the single questions (or to a cluster of questions) and the overall section score obtained by the participants. ‡ the answer to this question was considered in the evaluation of both the knowledge and the behaviour score.
